# Elevated acetate kinase (ackA) gene expression, activity, and biofilm formation observed in methicillin-resistant strains of *Staphylococcus aureus* (MRSA)

**DOI:** 10.1186/s43141-023-00555-0

**Published:** 2023-10-13

**Authors:** Subbarayudu Suthi, A. Mounika, Venkata Gurunadha Krishna Sarma Potukuchi

**Affiliations:** Microbial Genetics Laboratory, Department of Biotechnology, Sri Venkateswara Institute of Medical Sciences, Alipiri Road, Tirupati, 517501 Andhra Pradesh India

**Keywords:** Multidrug-resistant *Staphylococcus aureus*, Acetate kinase, Biofilm, Acetate, MRSA

## Abstract

**Background:**

*Staphylococcus aureus* spreads its infections through biofilms. This usually happens in the stationary phase of *S. aureus* growth where it utilizes accumulated acetate as a carbon source via the phosphotrans-acetylase-acetate kinase (Pta-Ack) pathway. In which acetate kinase (ackA) catalyzes the substrate-level phosphorylation, a vital secondary energy-yielding pathway that promotes biofilms formation aids bacterium survival in hostile environments. In this study, we describe the cloning, sequencing, and expression of *S. aureus* ackA gene. The expression analysis of ackA gene in methicillin-resistant strains of *S. aureus* (MRSA) correlates with ackA activity and biofilm units. The uniqueness of ackA was analyzed by using in silico methods.

**Results:**

Elevated ackA gene expression was observed in MRSA strains, which correlates with increased ackA activity and biofilm units, explaining ackA role in MRSA growth and pathogenicity. The pure recombinant acetate kinase showed a molecular weight of 44 kDa, with enzyme activity of 3.35 ± 0.05 μM/ml/min. The presence of ACKA-1, ACKA-2 sites, one ATP, and five serine/threonine-protein kinase sites in the ackA gene (KC954623.1) indicated that acetyl phosphate production is strongly controlled. The comparative structural analysis of *S. aureus* ackA with ackA structures of *Mycobacterium avium* (3P4I) and *Salmonella typhimurium* (3SLC) exhibited variations as indicated by the RMSD values 1.877 Å and 2.141 Å respectively, explaining why ackA functions are differently placed in bacteria, concurring its involvement in *S. aureus* pathogenesis.

**Conclusions:**

Overall findings of this study highlight the correlation of ackA expression profoundly increases survival capacity through biofilm formation, which is a pathogenic factor in MRSA and plays a pivotal role in infection spreading.

## Introduction

*Staphylococcus aureus* is a Gram-positive, facultative anaerobe human pathogen that is the leading cause of nosocomial and community-acquired infections [1–3]. This human pathogen can adapt and colonize on both biotic and abiotic surfaces and it can infect several anatomical sites in the human body via adaptive metabolism and biofilm formation [4–6]. The bacterium’s adaptability is based on its ability to detect and utilize nutrients from a variety of sources, as well as respond effectively to rapid environmental conditions. This is accomplished by modulating the expression of genes involved in several metabolic pathways, which effects the expression of virulence factors, and biofilm formation [7, 8].

Biofilms are surface-associated multicellular communities in which bacteria are embedded in a self-produced extracellular polymeric substance (EPS) which is mainly composed of polysaccharides, proteins, lipids, and nucleic acids. Biofilm formation occurs during the stationary phase of growth and is aided by the secretion of many virulence factors, cell wall-associated adherence proteins such as protein A, and fibronectin-binding proteins that are important for colonization, nutrient acquisition, tissue invasion, and dodging of host defenses [9–13].

Ever-increasing rates of staphylococci infections, both community- and hospital-acquired strains, are often rising across the globe [14–16]. However, the acquisition of multidrug resistance and an increased rate of biofilm formation in *S. aureus* has posed difficulties in the treatment of the infections caused by these strains, particularly, community-associated methicillin-resistant *S. aureus* (CA-MRSA) that have resulted in the rapid spread of infections across the globe, causing significant morbidity, mortality, and economic loss [17–22]. The rapidity with which this pathogen spreads its infection from one human host to another is through the formation of biofilms, which not only helps the pathogen escape the harsh environment caused by antibiotic treatment but also paves the way for newer infections [23–25], and elevated biofilm formation was noted in MRSA [6, 12, 25–27].

*S. aureus* catabolizes carbohydrates primarily through the pentose phosphate and glycolytic pathways, generating pyruvate and ATP [8, 28, 29], which are largely dependent on redox status. Under anaerobic growth, pyruvate is reduced to lactic acid, while in aerobic growth, pyruvate undergoes oxidative decarboxylation to generate acetyl-coenzyme A [30–32]. This acetyl-CoA is converted into acetyl phosphate and is used in the substrate-level phosphorylation through the phosphotrans-acetylase-acetate kinase (Pta-AckA) pathway to generate acetate and ATP. An excess amount of acetate excretes into the culture medium until the concentration of glucose decreases to a level at which it can no longer sustain rapid growth. The departure from the exponential phase of growth increases the utilization of acetate in energy generation and biofilm formation [32–37]. In the absence of oxygen, *S. aureus* does not induce the full tricarboxylic acid (TCA) cycle [8, 29, 33, 34, 38]; thus, ATP must come from substrate phosphorylation of acetate and acetyl phosphate (acP) via the Pta-AckA pathway [33, 39, 40]. Acetate kinase catalyzes the reversible magnesium-dependent phosphorylation of acetate using ATP as a phosphate donor and plays a significant role in regulatory phosphorylation reactions via acetyl phosphate [31, 33, 36, 39, 41]. Previous evidence suggests that catabolism of acetate and acetyl phosphate functions as global signals involved in host–pathogen interactions, and biofilm formation is associated with alterations in the redox status and repression of TCA cycle activity [33, 36, 40–42]. Acetate generation and its utilization in bacteria are uniquely placed and it depends purely on the growth conditions and environment; thus, the acetate kinase structure and functions are also unique to the organism [43–45]. This enzyme’s uniqueness in each organism, particularly in pathogenic bacteria, aids its survival in a variety of clinical settings [5, 43–47]. Since acetate kinase is important in both catabolism and anabolism in *S. aureus* and the absence of acetate kinase in human beings helps the spread of infections caused by multidrug-resistant strains, these make this enzyme all the more important in view rapid occurrence of MRSA strains [41, 48, 49]. This drug resistance in *S. aureus* is correlated with elevated acetyl phosphate formation [42]; given the metabolic importance of acetate kinase and its subtle role in the survival and pathogenesis of *S. aureus*, the present study is aimed to characterize the acetate kinase gene and its expression in multidrug-resistant strains of *Staphylococcus aureus* with an emphasis on methicillin-resistant strains of *S. aureus* (MRSA) and its association in the biofilm formation.

## Methods

### Bacterial strains growth and culture conditions

*Staphylococcus aureus* ATCC12600 and multidrug-resistant strains of *S. aureus* (LMV-1, 2, 3, 4, 5, 6 isolated from Local Milk Vendor and D-1, 2, and 4 isolated from Dairy herd) were grown in Baird Parker medium. These strains were also plated in Muller Hilton agar plates with ampicillin, oxacillin, and penicillin. The methicillin-resistant strains of *S. aureus* LMV 3–5 (MRSA) showed resistance to ampicillin, oxacillin, and penicillin with the conspicuous presence of *mec A* gene [12, 50, 51]. The cultures were confirmed by Gram staining, coagulase test, and catalase tests. A single isolated colony was inoculated in both Luria–Bertani (LB) and Brain heart infusion (BHI) broths and grown overnight at 37 °C. The grown cultures were used for the isolation of cytosolic fraction, characterization of acetate kinase, isolation of chromosomal DNA, and total RNA.

### Acetate kinase (ackA) gene amplification and sequencing

Overnight grown culture of *S. aureus* ATCC12600 was used for the isolation of genomic DNA [51] and the isolated chromosomal DNA was used for the amplification of ackA (Table 1). The PCR products were electro eluted and the sequence was performed by dye terminating method at commercial service (MWG Biotech India Ltd). The obtained sequence was analyzed and deposited in GenBank (accession number: KC954623.1). For expressing the ackA gene, new primers containing the sites for restriction enzymes Sal I and Hind III were used (Table 1). The PCR products were electrophoresed in 1.2% agarose gel and were electroeluted from the agarose gel [12, 52].

**Table 1:** Primers for the amplification of ackA gene from the *S. aureus* ATCC12600 and used in the present study

Primers	PCR conditions	PCR product size in Kbp
**A: For primary sequencing of *ackA* gene** **Forward Primer:** 5′ATGTCAAAATTAATC 3′ **Reverse Primer:** 5′TTATTTTAGACCACCG 3′	one cycle 5 min at 94 °C, 35 cycles 50 s at 94 °C, 45 s at 36.5 °C 2 min at 72 °C, and a final hold 10 min at 72 °C	1.2
**B: For cloning and expression of *ackA* gene** **Forward Primer:** 5′TTACTTGTCGACATGTCAAAATT3′ **Reverse Primer:** 5′AAAAAAAAGCTTTTATTTTAGACC3′	one cycle 5 min at 94 °C, 35 cycles 50 s at 94 °C, 45 s at 36.5 °C 2 min at 72 °C, and a final hold 10 min at 72 °C	1.2

### Cloning, expression, and characterization of acetate kinase (ackA) gene

The amplified PCR product was electroeluted from agarose gel and digested with Sal I and Hind III restriction endonucleases. The resultant digested PCR product was ligated with pQE 30 (QIAGEN, Valencia, CA) digested with Sal I and Hind III, and transformed into *E. coli* DH5α [51, 52]. The positive recombinant clones were identified by PCR and sequencing. The clone PSackA containing the ackA gene was grown in LB medium containing ampicillin (100 μg/ ml) at 37 °C, and expression of the His-6-tagged acetate kinase was induced with 1 mM IPTG. The recombinant protein was purified using a nickel-metal chelate agarose column and was analyzed in 10% SDS-PAGE [12, 51, 52].

### Acetate kinase assay

Acetate kinase (ackA) is present in the cytoplasm of bacteria; therefore, cytosolic fraction was isolated from the overnight grown cultures of *S. aureus* strains [51]. The assay mixture 0.5 ml contained 100 mM Tris-HC1 buffer pH 7.3, 2 mM potassium acetate, 1.5 mM ATP, 2 mM MgC1_2_, 2 mM phosphoenolpyruvate, 0.4 mM NADH, 5 units of pyruvate kinase (PK), and 10 units of lactate dehydrogenase (LDH). Assays were initiated by the addition of either the cytosolic fraction or pure recombinant ackA, the reaction was monitored by taking optical density at 340 nm against blank, i.e., without a cytosolic fraction or pure recombinant ackA, and the enzyme activity was expressed as μM of NADH formed for ml per minute [36, 52, 53].

### Real-time PCR

MEDOX-Easy™ Spin column total RNA mini preps kit was used to isolate total RNA from all the strains of *S. aureus* included in the present study, and cDNA was generated using high capacity cDNA reverse transcription kit (Applied Biosystems). Reverse transcription was performed in a thermocycler standardized at 25 °C for 10 min, 37 °C for 120 min, and 85 °C for 5 min, and thus formed cDNA was used as a template in quantitative real-time PCR (qPCR). The qPCR was executed in ABI 7300 with an SYBR select master mix for 45 cycles. The expression of the ackA gene in different strains of *S. aureus* was measured using DNA gyrase expression as an endogenous control (Table 2). The expression levels were calculated by using the (2^−ΔΔCt^) method [12, 54].

**Table 2:** Quantitative Real time PCR primers for the studying of ack A gene expression in different strains of *S. aureus*

Genes	Primers	qPCR conditions
DNA gyrase	**Forward Primer** CGATTGCTCTAGTAAAAGTCCTGAAG **Reverse Primer** GTAGACCCCCCGGCAGAGT	50 °C for 2 min; 95 °C for 10 min; 45 cycles of 95 °C for 15 s and 60 °C for 1 min
ackA	**Forward Primer** CGCTTCACAACCCAGCTAACTT **Reverse Primer** CGCCACATGAGGGATATTCG	50 °C for 2 min; 95 °C for 10 min; 45 cycles of 95 °C for 15 s and 60 °C for 1 min

### Evaluation of biofilm units (BU)

BU was evaluated in 96-well flat-bottomed Nunclon microtiter polystyrene plates as illustrated earlier [6, 12]. All strains were grown up to the mid-log phase in both BHI and LB broths. The grown mid-log phase cultures were diluted in BHI broth and 200 μl of diluted cells was added to individual wells and incubated for 1 day at 37 °C, followed by being washed softly with 0.1 M phosphate-buffered saline pH 7.4 and air-dried. The bound biofilms were stained with 0.4% crystal violet. Absorbance was recorded at 570 nm for the calculation of A_biofilm_. Simultaneously A_growth_ was determined for cultures grown in LB and BHI broths under static conditions at 37 °C for 24 h by taking absorbance at 570 nm. The BU was calculated by using the following formula: BU = A_biofilm_/A_growth_ [6, 12].

### In silico analysis of ackA

ackA amino acid sequence of *S. aureus* ATCC12600 was analyzed by using the NCBI-BLAST and Clustal X tool. The multiple sequence alignment was performed between *S. aureus* ackA sequence with *Mycobacterium avium*, *Salmonella typhimurium*, *Methanosarcina thermophila*, and *E. coli* using ClustalX software. The crystal structure of *S. aureus* ackA was not available in the PDB; therefore, *S. aureus* ackA structure was built from its sequence (KC954623.1) by using the online SWISS-MODEL tool. The structural comparisons of *S. aureus* ackA structure with other bacterial ackA structures were carried out using PyMOL. An alignment of superimposed structures and similarities were predicted and the differences in the structures were expressed in terms of root means square deviation (RMSD) values for the *Mycobacterium avium* (3P4I) and *Salmonella typhimurium* (3SLC) [55].

### Statistical analysis

All the experiments were performed AQ2 two times (*n* = 2) and all the values were given as mean ± standard deviation of the mean (SD). Statistics analysis was evaluated using two-way ANOVA with *p*-value < 0.05.

## Results

### Characterization of *S. aureus* acetate kinase (ackA)

**Fig. 1:**
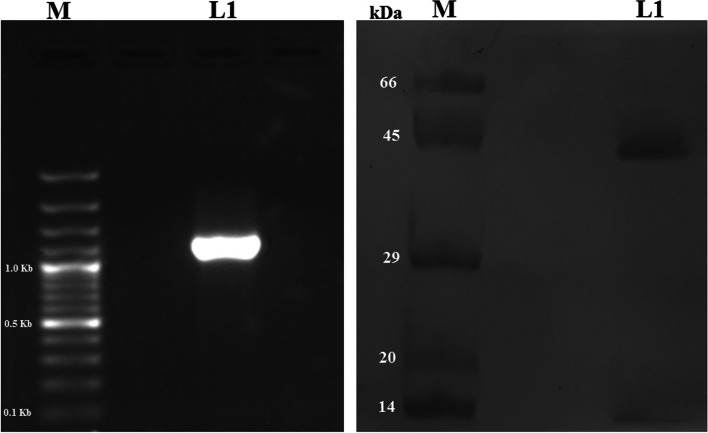
PCR amplification, expression, and purification of *S. aureus* ATCC 12600 ackA (**a**) polymerase chain reaction of ackA from genomic DNA of *S. aureus* ATCC 12600: where Lane M is 100 bp DNA ladder, Lane 1 is PCR product of ackA gene (1.2 Kb) (**b**) 10% SDS-PAGE analysis of expressed recombinant ackA in *E. coli* DH5α: Lane M is medium range protein molecular weight marker obtained from Bangalore Genei pvt ltd India, Lane 1 is purified recombinant ackA of clone PSackA induced with 1 mM IPTG showing a molecular weight of 44 kDa

*S. aureus* ATCC12600 acetate kinase (ackA) gene was PCR amplified (Fig. 1a) and the resultant PCR product of ackA (1.2 Kb) gene was ligated into Sal I and Hind III sites of pQE 30 vector and transformed into *E. coli* DH5α; the positive recombinant clones were identified and confirmed by PCR and sequencing (Table 1) The resultant clone was named PS ackA and the sequence was submitted to GenBank (Accession number: KC954623.1). The *ackA* gene expression was successfully induced with 1 mM IPTG in PS ackA clone. The recombinant ackA was purified by passing through the nickel-metal chelate affinity column. The purity of the recombinant ackA was assessed on 10% SDS-PAGE, and the results showed a single band with a molecular weight of 44 KD corresponding to the insert cloned (Fig. 1b). The *ackA* gene sequence when BLAST searched resembled all the *ackA* gene sequences reported for other strains of *S. aureus* in the database.

**Fig. 2:**
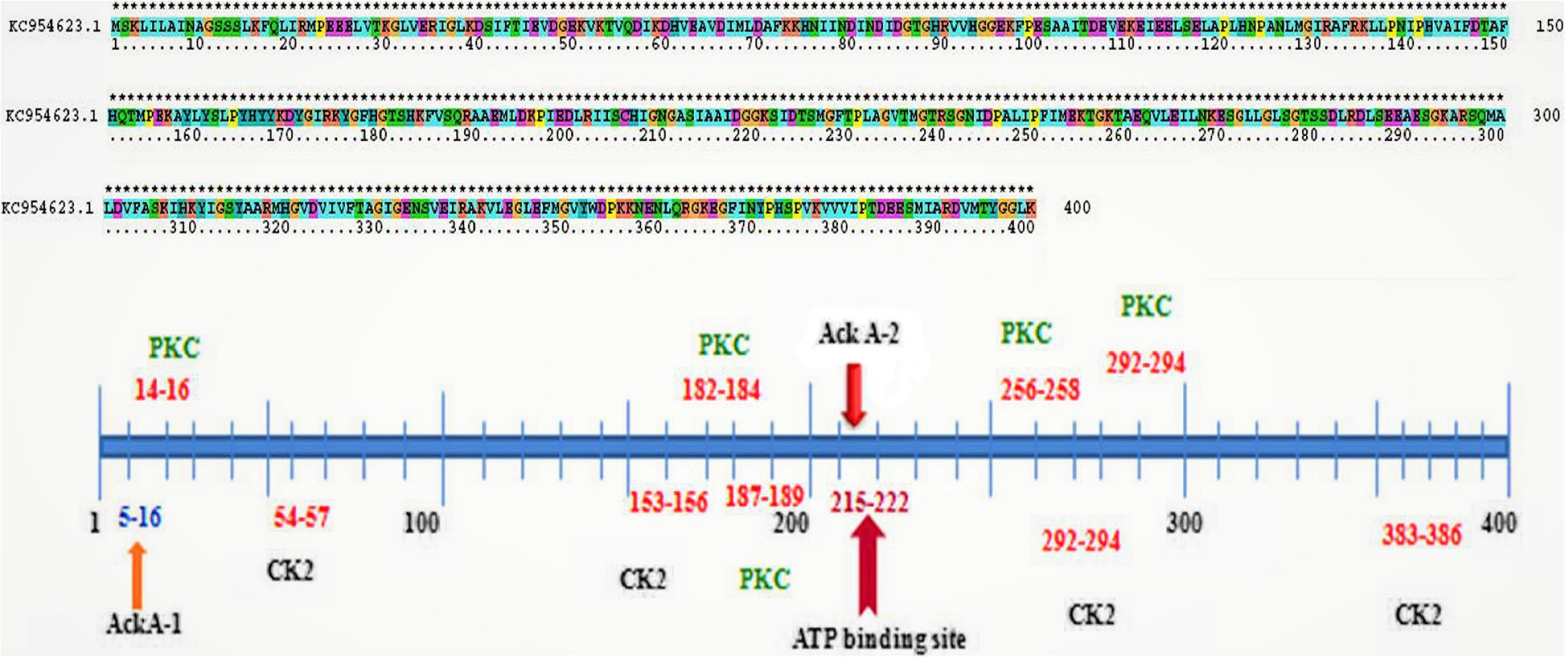
*S. aureus* ATCC12600 acetate kinase amino acid sequence alignment and PROSITE results showing ATP binding site, 5 serine-threonine kinase phosphorylation sites

The ackA amino acid sequence analysis (GenBank: KC954623.1) indicated the presence of (i) ACKA-1 (5–16 amino acids), (ii) ACKA-2 (215–222 amino acids), (iii) ATP binding site present inside the ACKA-2 domain, and (iv) interestingly, presence of five serine/threonine phosphorylation sites (Fig. 2). This organization of ackA explains that in *S. aureus*, the functioning of ackA is regulated by serine/threonine protein kinase.

### *S. aureus* ackA structural analysis

**Fig. 3:**
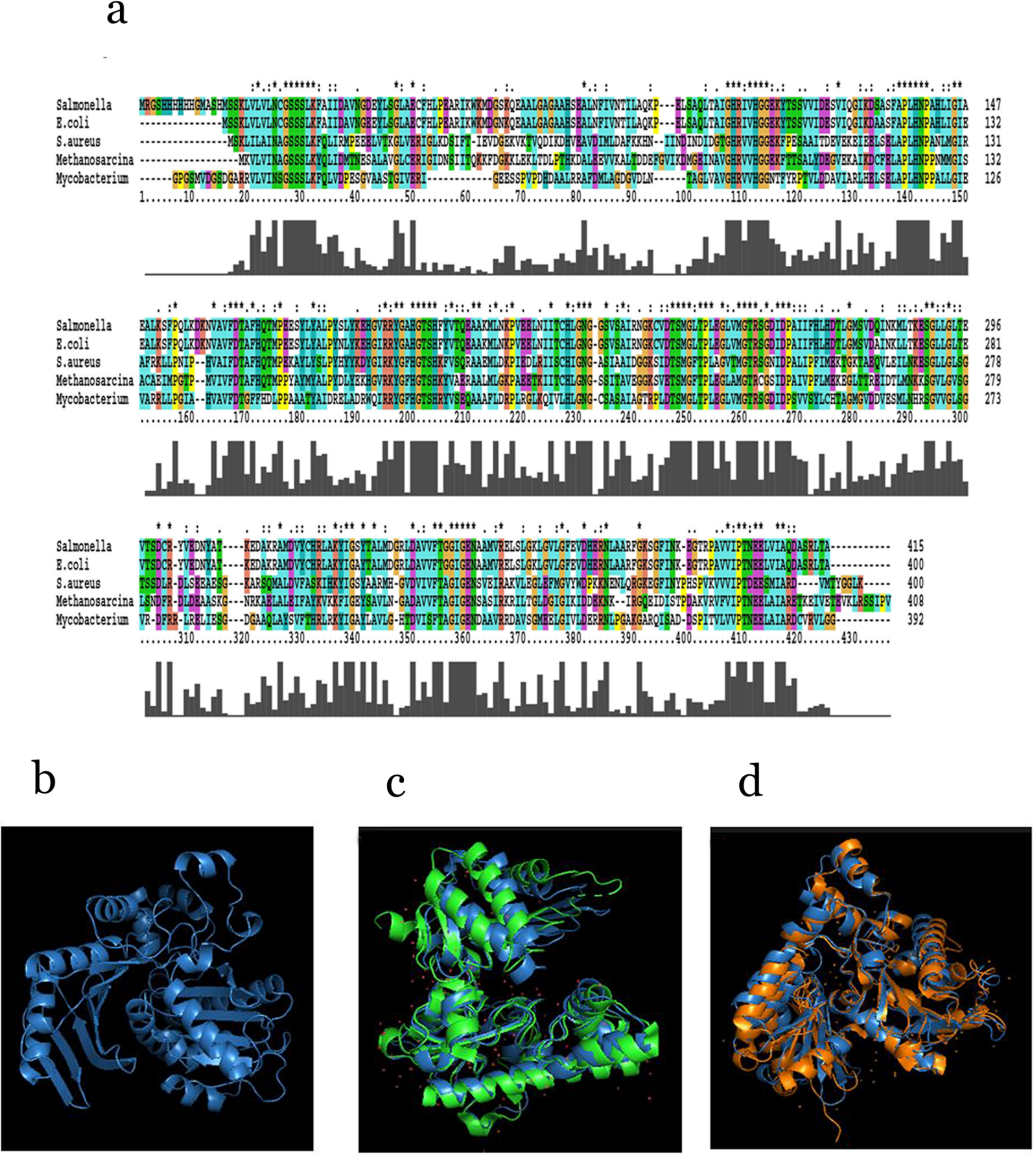
Multiple sequence alignment and structural analysis of *S. aureus* ATCC12600 ackA. **a** Multiple sequence alignment of ackA amino acid sequence of *S. aureus* ATCC12600 with *Mycobacterium avium*, Salmonella *typhimurium*, *Methanosarcina thermophila*, and *E. coli ackA.* **b** *S. aureus* ATCC12600 ackA structure built by using SWISS-MODEL and expressed in PyMOL program (blue). **c** PyMOL visualization and structural superimposition of ackA structure of *S. aureus* ATCC 12600 with *Mycobacterium avium* ackA green (3P4I) exhibited 1.877 Å RMSD value. **d** PyMOL visualization structural superimposition of ackA structure of *S. aureus* ATCC 12600 with *Salmonella typhimurium* ackA light orange (3SLC) exhibited 2.141 Å RMSD value

In multiple sequence alignment of ackA protein sequence showed conspicuous variations with *Mycobacterium avium*, *Salmonella typhimurium*, *Methanosarcina thermophila*, and *E. coli* (Fig. 3a). The *S. aureus* ackA structure built using the SWISS-MODEL (Fig. 3b) when compared with the ackA structures of *Mycobacterium avium* (PDB: 3P4I) and *Salmonella typhimurium* (PDB: 3SLC) showed very low structural homology as indicated from the RMSD values 1.877 Å, 2.141 Å respectively (Fig. 3c, d). The identical regions were distributed randomly throughout the alignment. These results indicate the uniqueness of the *S. aureus* ackA structure.

### Enzyme kinetics

**Fig. 4:**
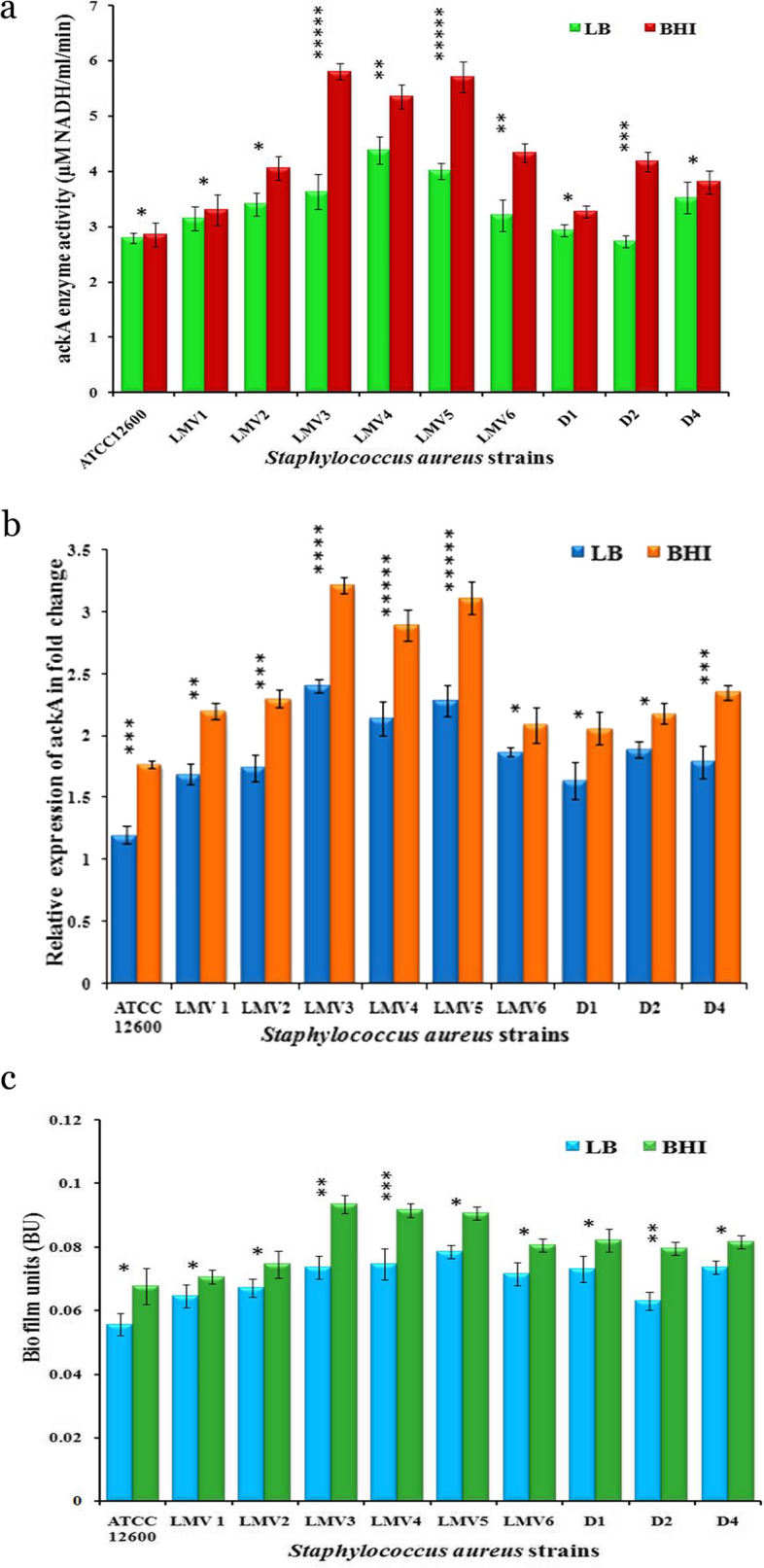
Analysis of ackA enzyme activity, expression, and biofilm units in different strains of *S. aureus* grown in LB and BHI broths. **a** ackA activity in all strains of *S. aureus* and the activity was expressed as μM of NADH formed for ml per minute. **b** Quantitative real-time PCR analysis of ackA gene in different strains of *S. aureus.* **c** The in vitro estimation of BU in different strains of *S. aureus* and BU was calculated by using the following formula: BU = A_biofilm_/A_growth_. Two-way ANOVA statistical significance: *p > 0.05, **p ≤ 0.05, ***p ≤ 0.01, ****p ≤ 0.001, and *****p ≤ 0.0001

Acetate kinase activity was identified in the cytosolic fraction of *S. aureus* ATCC12600 (2.85 ± 0.05 μM NADH/min/ml), while the recombinant ackA exhibited an activity of 3.35 ± 0.05 μM NADH/ml/min which was close to the native *S. aureus* ATCC12600. Distinct differences were noted when *S. aureus* ackA activity was compared to that of other prokaryotes (Table 3), indicating the uniqueness of this activity in *S. aureus*, which allows them to survive in a variety of environments, particularly in multidrug-resistant strains. The ackA activities were determined in the cytosolic fraction of multidrug-resistant strains of *S. aureus* grown in LB and BHI broths, which indicated higher ackA activity was observed in the multidrug-resistant strains of *S. aureus* grown in BHI broth compared to LB broth. Although multidrug-resistant strains showed higher ackA activity compared to drug-sensitive *S. aureus* ATCC12600 strain, the ackA activity in methicillin-resistant strains of *S. aureus* (LMV 3–5) (MRSA) strains was highly pronounced (Fig. 4a). These findings indicate that acetate requirement is high in multidrug-resistant *S. aureus* especially in MRSA.

**Table 3:** Acetate kinase (ackA) activity in various microorganisms

**Organism**	**ackA Activity** (μm/min/ mg or μm/min/ ml)	**References**
*Lactobacillus sanfranciscensis*	14.8	Knorr, R et al. 2001 [56]
*E. coli* JM109	2.1	Knorr, R et al. 2001 [56]
*Mycobacterium tuberculosis*	0.948	Rücker et al. 2015 [57]
*Desulfovibrio piger* Vib-7	1.52	Kushkevych IV. 2014 [58]
*Desulfomicrobiu* sp. Rod-9	0.46	Kushkevych IV. 2014 [58]
*Shewanella* sp. AS-11	56,640 U	Tang et al.2012 [59]
*Clostridium ultunense*	0.96	Anna Schnürer et al. 1997 [60]
*Clostridium acetobutylicurn*	401 U	Winzer et al. 1997 [61]
*Acholeplasma laidlawii*	268 u	Kahane I and A Muhlrad. 1979 [62]
*Methanosarcina thermophila*	196.4	Aceti and Ferry. 1988 [53]
*Salmonella typhimurium*	20.6	Fox and Roseman. 1986 [63]
*Staphylococcus aureus* ATCC 12600	3.35	This study

### Biofilm assay

Biofilm units (BU) were estimated for all the strains of *S. aureus* used in the present study grown in LB and in BHI broths. Elevated BU was observed in multidrug-resistant strains of *S. aureus* grown in BHI broth compared to all the *S. aureus* strains grown in LB broth. Interestingly, MRSA strains exhibited much higher BU compared to all other strains used in the present study in both the broths (Fig. 4c). These findings explain that multidrug-resistant strains of *S. aureus* particularly MRSA strains are more pathogenic in nature.

### Quantification of ackA gene expression using qPCR

The relative expression of the ackA gene in drug-resistant strains of *S. aureus* grown in LB and BHI broths was quantified against DNA gyrase expression as an endogenous control. The high expression of ackA was observed in all the *S. aureus* strains grown in BHI broth compared to the strains grown in LB broth. Among them, multidrug-resistant strains of *S. aureus* showed 1.37-folds of higher *ackA* gene expression compared to drug-sensitive *S. aureus* ATCC12600. Between multidrug-resistant strains of *S. aureus*, MRSA strains demonstrated 0.88-fold elevated expression (Fig. 4b). This elevated ackA gene expression aptly correlated with increased BU in multidrug-resistant strains of *S. aureus*, particularly MRSA strains. All these results explain that the requirement of acetate and acetyl phosphate drives the formation of higher BU in multidrug-resistant strains of *S. aureus* and in MRSA strains; thereby, ackA gene expression is involved in the pathogenesis of *S. aureus*, especially MRSA.

## Discussion

*Staphylococcus aureus* is a versatile pathogen that can survive in diverse niches and its versatility is based on its capacity to obtain and utilize nutrients from a variety of sources and respond by modifying gene expression; therefore, the metabolic signals encountered by bacteria not only aid in their survival in harsh conditions, but also play an important role in pathogenesis [5, 7, 14, 20, 47]. During growth under aerobic conditions, the end product of the glycolytic pathway, pyruvate, is decarboxylated to acetyl-coenzyme A. This acetyl-coenzyme A is converted to acetyl phosphate that is used to produce ATP and acetate and excess acetate is excreted into the culture medium during the exponential phase of growth, yet during the stationary phase of growth, when nutrient availability is minimal, *S. aureus* utilizes accumulated acetate as a carbon source via acetate kinase (ackA) [33, 36, 37]. In this process, acetate kinase is a key enzyme responsible for the dephosphorylation of acetyl phosphate with the concomitant production of acetate and ATP during anaerobic growth [33, 36, 64, 65]; the present study results concur with these findings. We have observed elevated ackA gene expression and enzyme activity in multidrug-resistant strains of *S. aureus* grown in BHI broth; interestingly, in MRSA strains, this was more noticeable (Fig. 4a, b).

**Fig. 5:**
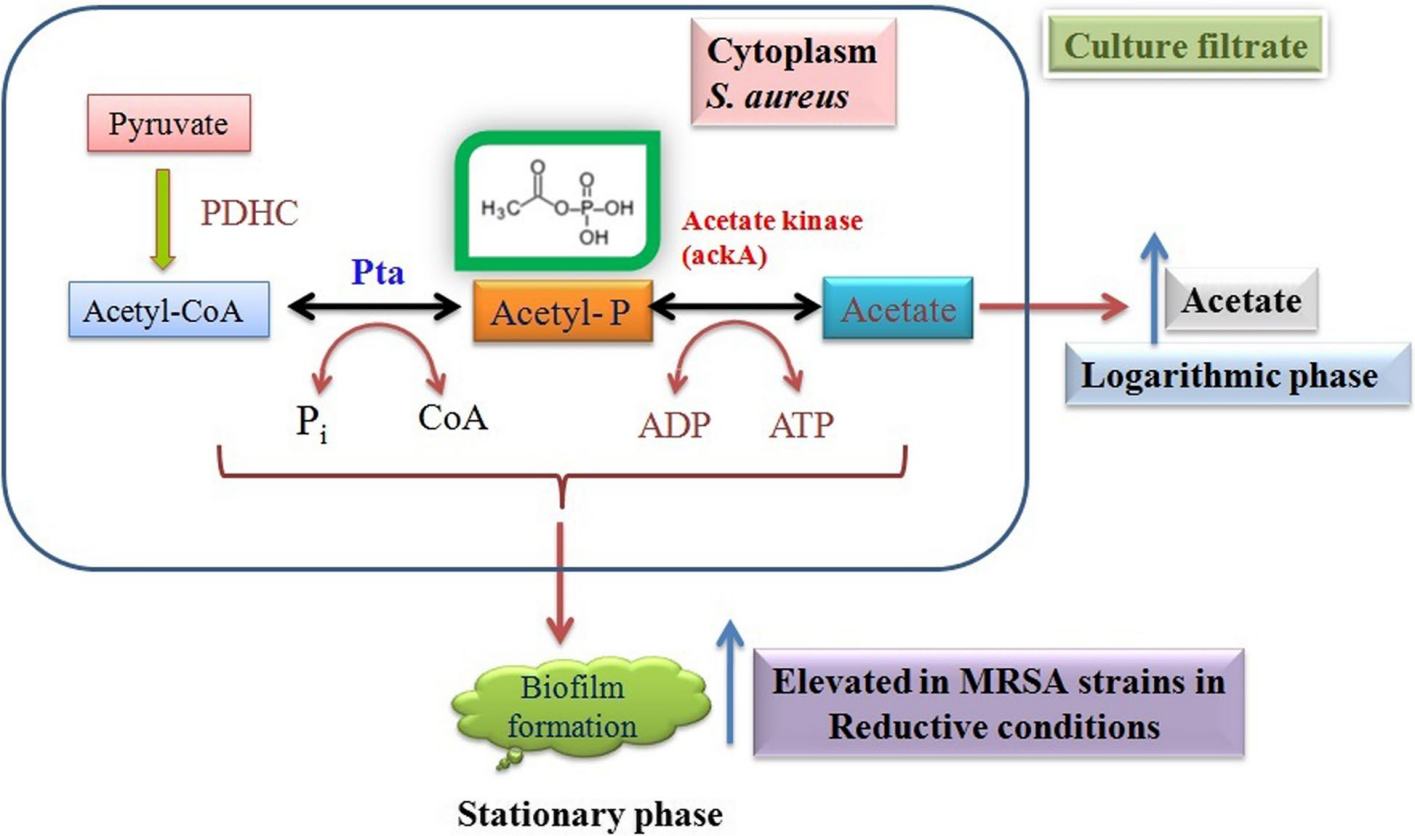
In MRSA under reductive conditions elevated ackA activity increases biofilm units

The gene encoding acetate kinase (ackA) was cloned and expressed in the *E. coli* DH5α and the pure recombinant ackA exhibited similar enzyme activity to that of native ackA (Fig. 1). The sequence analysis revealed that ackA of *S. aureus* was showing a distinct presence of S and T phosphorylating sites and an ATP binding site; this explains that ackA may be regulated by serine/threonine kinase [65]. The uniqueness of ackA function in various organisms is very much noted (Table 3); we also observed subtle structural variations between ackA of *S. aureus* and *Mycobacterium avium* (PDB: 3P4I), and *Salmonella typhimurium* (PDB:3SLC) (Fig. 3), which are associated with ackA function (Table 3) explaining the ackA functions are exclusively placed in bacteria [45, 46, 48, 66]. The elevated ackA activity and biofilm formation observed in MRSA strains indicate the ackA involvement in the biofilm formation (Fig. 4c) [23, 42–44]. Earlier studies from our laboratory showed elevated biofilm formation in MDR strains of *S. aureus* grown in BHI broth and more particularly in MRSA strains [12, 27], and the present study results also reconfirm (Fig. 4c). The interesting fact increased ackA expression and activity observed in MDR strains and in particular MRSA strains [5–8, 11, 40, 42] largely reveal that the acetyl phosphate whose forms changed due to acetate kinase activity participated in the anabolic biosynthesis of energy generation (Fig. 5). These findings suggest that higher biosynthesis means elevated rate of biofilm formation, which is a pathogenic factor of *S. aureus*; therefore, increased acetate kinase activity made greater availability of acetate and ATP, which actively participated in synthesis, leading to biofilm formation (Fig. 5) [33, 39, 40, 42, 51, 65, 66]. Thus, substrate-level phosphorylation remains a conserved and crucial mechanism in which gene disruption leads to a lethal condition [36, 39, 41, 44]. The findings of the present study indicate elevated ackA activity and expression correlated with increased biofilm units in multidrug-resistant strains of *S. aureus* explaining the acetate build-up profoundly increases the pathogenicity in MDR strains of *S. aureus* in particular MRSA (Fig. 5) [40, 42, 66].

## Conclusion

Acetate kinase is a central enzyme that is essential for the survival of *S. aureus* in different environmental conditions. In the current study, we have observed elevated acetate kinase activity, expression, and increased BU in multi-drug resistant strains of *S. aureus,* especially in MRSA strains, which indicates the drug resistance character elevates pathogenicity in multidrug-resistant strains of *S. aureus* and MRSA. Further, distinct structural differences were noted between ackA of *S. aureus* and other bacteria. All these findings explain the acetate build-up profoundly increases the survival capacity and pathogenicity in drug-resistant strains of *S. aureus* via biofilm formation which is the pathogenic factor in MRSA.

## Data Availability

The data presented in the manuscript are available with the corresponding author who can be contacted at a reasonable time. Some of the data that is Cloning of ackA available with Gen bank (accession no: KC954623.1).

## Abbreviations

ackA: Acetate kinase
acCoA: Acetyl-coenzyme A
Acetyl-P: Acetyl phosphate
ATCC: American Type Culture Collection
BHI: Brain heart infusion
BLAST: Basic Local Alignment Search Tool
BU: Biofilm units
cDNA: Complimentary DNA
D: Dairy herd
CA-MRSA: Community-associated methicillin-resistant *S. aureus*
EDTA: Ethylene diamine tetra acetic acid
EPS: Extracellular polymeric substance
IPTG: Isopropyl β-d-1-thiogalactopyranoside
LB: Luria*-*Bertani
LMV: Local Milk Vendor
MDR: Multidrug resistant
MRSA: Methicillin-resistant *Staphylococcus aureus*
PDB: Protein data bank
PCR: Polymerase chain reaction
Pta-AckA: Phosphotrans-acetylase-acetate kinase
pQE 30: Plasmid QIAGEN 30
SDS-PAGE: Sodium dodecyl sulphate–polyacrylamide gel electrophoresis
RMSD: Root means square deviation
TCA: Tricarboxylic acid cycle
